# 10-HDA attenuates inflammatory responses in LPS-activated RAW264.7 cells *in vitro* via the NUR77/NF-κB pathways and improving mitochondrial functions

**DOI:** 10.3389/fphar.2026.1833265

**Published:** 2026-07-01

**Authors:** Kangwei Zhang, Zhen Zhou, Haoxiang Ou, Xiaoli Fu, Jiaqi Xiao, Xinyi Pan, Long Wang, Bo Xu, Yanting Su, Mingjie Wei, Zhenwang Zhang, Liqiong Huang, Yongfen Bao, Shigang Shan

**Affiliations:** 1 School of Stomatology and Ophthalmology, Xianning Medical College, Hubei University of Science and Technology, Xianning, Hubei, China; 2 School of Pharmacy, Xianning Medical College, Hubei University of Science and Technology, Xianning, Hubei, China; 3 School of Basic Medical Sciences, Xianning Medical College, Hubei University of Science and Technology, Xianning, Hubei, China; 4 Department of Obstetrics and Gynecology, Xianning Maternal and Child Health Hospital, Xianning, Hubei, China

**Keywords:** 10-HDA, inflammation, macrophage polarization, mitochondrial function, Nur77

## Abstract

**Background:**

Inflammation is implicated in the pathogenesis of various diseases, including cancer, diabetes, and diseases caused by bacterial infections. Macrophages, as key regulators of inflammatory responses, can polarize into distinct phenotypes such as pro-inflammatory M1 and anti-inflammatory M2 macrophages. Trans-10-hydroxy-2-decenoic acid (10-HDA), a bioactive component of royal jelly (RJ), exhibits potent anti-inflammatory properties; however, its influence on macrophage polarization remains poorly understood.

**Methods:**

10-HDA was used to uncover the mechanisms for LPS-induced inflammatory response in RAW264.7 cells. CCK8 assay, ELISA assay, western blot analysis, real-time PCR, immunofluorescence assay were performed to evaluate the protective effects of 10-HDA.

**Results:**

Our findings demonstrated that 10-HDA treatment upregulated M2 macrophage markers, enhanced the secretion of anti-inflammatory cytokines, and modulated multiple signaling pathways. Mechanistically, 10-HDA exerted its anti-inflammatory effects by targeting the NUR77/NF-κB pathway, suppressing the expression of inducible nitric oxide synthase (iNOS) while promoting the anti-inflammatory marker CD206. Additionally, 10-HDA significantly inhibited the production of pro-inflammatory cytokines, including IL-6, IL-1β, and TNF-α. Furthermore, 10-HDA influenced mitochondrial metabolism, suggesting a protective role in maintaining mitochondrial function.

**Conclusion:**

These results indicate that 10-HDA promotes M2 macrophage polarization through the NUR77/NF-κB signaling pathway and by mitigating mitochondrial dysfunction, highlighting its potential role in modulating innate immunity and its potential application in anti-inflammatory therapy.

## 1 Introduction

Unlike the acute, transient inflammation that defends against infections, chronic low-grade inflammation is a sustained, non-resolving systemic state that does not necessarily involve classic pathogen-triggered macrophage effector phases (Xu et al., 2024). This low-grade inflammatory status is now recognized as a critical pathological basis for multiple chronic diseases. For instance, persistent production of pro-inflammatory mediators—such as cytokines and chemokines—plays a deleterious role in the progression of rheumatoid arthritis (Demoruelle et al., 2014), metabolic syndrome (Hotamisligil, 2006), atherosclerosis (Wolf and Ley, 2019), and various tumors (Coussens and Werb, 2002), by contributing to tissue damage, insulin resistance, plaque instability, and immune evasion, respectively. Therefore, the research and development of anti-inflammatory drugs is of great importance. At present, the commonly used clinical anti-inflammatory drugs are non-steroidal anti-inflammatory drugs (NSAIDs) and steroidal anti-inflammatory drugs. However, both classes have obvious adverse reactions. For example, long-term use of NSAIDs may cause gastrointestinal ulcers, bleeding, and renal impairment, while steroidal drugs are associated with immunosuppression, osteoporosis, hyperglycemia, and adrenal suppression. These adverse effects not only compromise patient compliance and quality of life but also limit their clinical application. In contrast, natural anti-inflammatory agents with fewer side effects have attracted increasing attention as promising alternatives.

RAW264.7 macrophages are widely used as a model for studying inflammatory responses with bi-directional pro-inflammatory and anti-inflammatory roles. M1-type RAW264.7 are activated by LPS, IFN-γ, and TNF-α, highly express iNOS, synthesize large quantities of NO and reactive oxygen species (ROS) intracellularly, and produce the pro-inflammatory factors IL-1β, IL-6, etc. (Xu et al., 2023). They can also be induced into the M2 phenotype, producing ARG1, CD206, and inflammation-suppressing factors IL-10 and TGF-β, etc. (Rőszer, 2015), and decreasing the expression of iNOS. Excessive inflammatory response or production of excessive ROS can damage tissue cells (Davalli et al., 2016).

RAW264.7 macrophages, a widely used murine macrophage cell line, are routinely cultured in high-glucose DMEM as recommended by ATCC. Although high-glucose culture conditions have been reported to promote M1-like polarization (e.g., via STAT-3/autophagy downregulation) and influence mitochondrial function, this concern is mitigated in the present study because all experimental groups (control, LPS, and 10-HDA treatment) were maintained under identical high-glucose DMEM conditions. Consequently, any potential baseline effect is equally distributed across groups and does not confound the comparative evaluation of 10-HDA’s anti-inflammatory activity. Moreover, the enhanced inflammatory signaling primed by high-glucose conditions may provide a more sensitive detection window for assessing the anti-inflammatory efficacy of test compounds, as previously observed in comparative studies between high-glucose DMEM and RPMI 1640.

Mitochondrial function is modulated by several rate-limiting enzymes, and their regulation is closely linked to macrophage polarization status. In pro-inflammatory (M1) macrophages, metabolic reprogramming toward glycolysis is accompanied by impaired oxidative phosphorylation and altered expression of mitochondrial enzymes. For instance, uncoupling protein 3 (UCP3), which reduces reactive oxygen species (ROS) production and limits hypoxia-induced damage (Brand and Esteves, 2005), is downregulated in M1 macrophages, but whether this change actively promotes or merely reflects the inflammatory state remains unclear. Similarly, cytochrome c (Cyc-C), a key electron transporter in the respiratory chain, enhances oxidative phosphorylation to support cellular energy demands (Sánchez-Pérez et al., 2023); however, its precise role in M1 versus M2 macrophages has not been systematically characterized. COX-2, an inducible enzyme that drives lipid peroxidation and prostaglandin synthesis, is rapidly upregulated upon LPS stimulation in RAW264.7 macrophages (Shah et al., 2024), yet how COX-2 coordinates with mitochondrial dysfunction during M1 polarization—and whether it is similarly involved in M2 cells—is largely unknown. Collectively, these examples illustrate that while certain mitochondrial enzymes are clearly altered in inflammatory macrophages, there is a lack of integrated information on how their polarization-specific expression patterns and functional consequences contribute to the divergent metabolic and inflammatory phenotypes of M1 versus M2 macrophages. Addressing this knowledge gap is essential for understanding the role of mitochondrial dysfunction in macrophage-mediated disorders.

RJ is a yellowish-white acidic secretion produced by worker bees. It has been reported to possess various biological activities; among these, its anti-inflammatory and immunomodulatory properties have been relatively well-documented in the literature, which forms the background of our current investigation. 10-HDA is one of the markers in RJ, which exists in very small quantities, and is a unique fatty acid found only in royal jelly, alias royal jelly acid (Honda et al., 2015). 10-HDA is a natural product. Although its safety profile has not been fully characterized, initial studies indicate low toxicity, which may be advantageous in the early stages of new drug development. In a recent study, 10-HDA was found to exert anti-inflammatory effects on LPS-stimulated RAW264.7 cells (Huang et al., 2023).

In previous studies, multiple effects of 10-HDA have been demonstrated. Hu Xiyi found that 10-HDA enhances glucose metabolism in type 2 diabetic mice on a high-fat diet through the PI3K/Akt/mTOR signaling pathway (Hu et al., 2022). Huang Shanshan found that 10-HDA also enhances immunity and alleviates inflammatory responses *in vivo* by modulating the NLRP3 inflammasome-mediated cleavage pathway (Huang et al., 2022a). In addition, 10-HDA has been reported to exhibit inhibitory effects against a variety of tumors (Tian et al., 2024; Saad Al Shehri et al., 2023; Albalawi et al., 2021; Lin et al., 2020; Botezan et al., 2023) and bacteria (Gao et al., 2022; Šedivá et al., 2018; Yang et al., 2018; Nazem et al., 2025); however, the mechanisms underlying these effects particularly the antimicrobial action are distinct from the direct immunomodulatory effects on macrophages that we aim to investigate. Moreover, most of these studies were conducted *in vivo*, where multiple cell types and tissue environments may contribute to or confound the observed activities, potentially influencing macrophage function indirectly.

In this experiment, we investigated the induction of LPS on RAW 264.7 *in vitro* in culture, established an inflammation model, and elucidated the mechanism of anti-inflammatory effect of 10-HDA on macrophage polarization. The results showed that 10-HDA attenuated LPS-induced M1 polarization and partially shifted the macrophage phenotype toward an M2-like state by affecting the expression of inflammatory cytokines, NUR77 and NF-κB pathways, and by decreasing the levels of COX-2 and iNOS in the LPS-induced inflammatory model, while attenuating the damage of inflammation to mitochondria.

## 2 Materials and methods

### 2.1 Main reagents and antibodies

10-HDA (purity ≥98%) was purchased from Sigma-Aldrich (St. Louis, MO, United States). Fetal bovine serum was purchased from Jiangsu Ecosay Biological Company. TransScript First-Strand cDNA Synthesis Kit and TransStart Top Green qPCR Kit were purchased from Transgen Biotech (Beijing, China). LPS, RIPA lysate, protease inhibitors were obtained from Sigma-Aldrich (St. Louis, Missouri, United States). BCA protein quantification kit, HRP-labeled sheep anti-rabbit secondary antibody, skimmed milk, triton solution, 4% paraformaldehyde, fluorescent secondary antibody, ultrasensitive chemiluminescent developer, and mitochondrial red fluorescent probe were purchased from Beyotime Biological Company (Shanghai, China). Enzyme-linked immunosorbent assay (ELISA) kits for determining mouse tumor necrosis factor-α (TNF-α), interleukin-1β (IL-1β), and interleukin-6 (IL-6) were purchased from Mabtech (Stockholm, Sweden). NUR77, Cyc, COX-2, UCP3, INOS, CD206, phosphorylated NF-κB (p-NF-κB), nuclear factor kappa B (NF-κB), β-catin, Inhibitor of NF-κB α (IκBα) primary antibody were purchased from Cell Signaling Technology (Beverly, Massachusetts, United States).

### 2.2 Cell culture and treatment

RAW264.7 cells were obtained from the American Type Culture Collection (ATCC, Manassas, VA). Cells were incubated at 37 °C in a humidified incubator using high-glucose DMEM(4.5 g/L or 25 mM glucose, Gibco) supplemented with 10% fetal bovine serum (FBS, Gibco) and 1% penicillin-streptomycin. Three groups were set up: a control group (vehicle control containing an equal amount of DMSO), an LPS group (500 ng/mL LPS treatment for 12 h or 24 h), and an administration group. In the administration group, cells were first treated with LPS (500 ng/mL) for 1 h, followed by co-incubation with 10-HDA (1, 3, or 5 mM) for an additional 12 h or 24 h (with LPS remaining in the medium throughout).

### 2.3 Cellular CCK-8 experiments

Cell viability was assessed using a Cell Counting Kit-8 (Dojindo, Kumamoto, Japan) following the manufacturer’s instructions. Briefly, 1 × 10^4^ cells/well were seeded into 96-well plates and treated as described in Section 2.2. After the indicated treatments, 90 μL of medium and 10 μL of CCK-8 solution were added to each well, followed by incubation at 37 °C for 2 h. Absorbance at 450 nm was measured using an Infinite M200 microplate reader (Tecan, Switzerland). Cell viability was calculated as (absorbance of treated cells/absorbance of control) × 100%.

### 2.4 Measurement of IL-1β, IL-6 and TNF-α

Cell treatment was performed as described in Section 2.3. Based on preliminary experiments showing that LPS-induced cytokine secretion reached a stable and significant level at 24 h, while changes at 12 h were minimal and not statistically different from controls (data not shown), we collected supernatants only after 24 h of incubation. The levels of TNF-α, IL-6, and IL-1β were measured using ELISA kits.

### 2.5 Detection of protein expression level by western blotting method

Cells were inoculated into 6-well plates and treated according to the order of control group, experimental group and drug administration group for 12 h or 24 h. After incubation, cells were lysed using RIPA lysis buffer, and then protein concentration was adjusted to be consistent using BCA Protein Quantification Kit, and denaturation was carried out for 10 min after adding ¼ of 5×Buffer. The proteins were then separated by gel electrophoresis and transferred to PVDF membrane for primary and secondary antibody incubation, and protein bands were visualized using an ultrasensitive chemiluminescence detection system, and images were processed with ImageJ.

### 2.6 Determination of mRNA levels

For qRT-PCR, RAW264.7 cells were seeded in six-well culture plates (5 × 10^5^/well) and cultured overnight. After various treatments, total RNA was isolated using RNeasy Mini extraction kit and then first-strand cDNA was synthesized using RT2 first Strand Kit following the manufacturer’s protocol. PCR amplification was performed using RT2 SYBR Green Fluor PCR Mastermix. Amplification was performed using an Applied Biosystems 7500 Real-Time PCR System. Oligonucleotide primers are listed in Table 1. From each sample 25 μL PCR product was used to run the RT2 Profiler PCR array according to the manufacturer’s protocol. The PCR thermal cycling programs were: denaturation at 95 °C for 10 min, followed by 40 cycles at 95 °C for 15 s, 60 °C for 1 min. Data analysis was performed using RT2 Profiler PCR array data analysis on line software (http://www.sabiosciences.com/pcrarraydataanalysis.php).

**TABLE 1:** Primer sequences for qRT-PCR.

Gene	Forward primer (5’-3’)	Reverse primer (5’-3’)
GAPDH	CCT​CGT​CCC​GTA​GAC​AAA​ATG	TGA​GGT​CAA​TGA​AGG​GGT​CGT
iNOS	CTG​TCG​CAG​CTC​CCT​ATC​TT	TCA​GGT​TCC​TGA​TCC​AAG​TGC
CD206	CAG​GAG​GAC​TGC​GTG​GTT​ATG	GGT​TTG​CAT​CAG​TGA​AGG​TGG
COX-2	GAA​ATA​TCA​GGT​CAT​TGG​TGG​AGA	ATG​CTC​CTG​CTT​GAG​TAT​GTC​G
IL-1β	AGG​CTC​CGA​GAT​GAA​CAA​CAA​A	GTG​CCG​TCT​TTC​ATT​ACA​CAG​GA

### 2.7 Immunofluorescence assay

Cells were inoculated on slides, according to the control group, the experimental group, the drug group after the end of the treatment, discard the medium, washed twice with PBS, add 4% paraformaldehyde fixed for 15 min, and then add the appropriate amount of 0.2% triton PBS solution placed at room temperature for 5 min, and then washed three times with PBS, each time for 5 min, and then add 5% skimmed milk closed 30min, closed After the end of the liquid was aspirated, add 5% skimmed milk prepared primary antibody solution placed in 4 °C shaking bed overnight, the next day after the first antibody was aspirated and washed three times with PBS, then add 5% skimmed milk prepared fluorescent secondary antibody solution placed on the shaking bed to avoid the light for 1h, the secondary antibody was washed three times at the end of the incubation, and then add DAPI solution and incubate in the dark for 5 min, and then washed three times with PBS to seal the film, and then put into the fluorescence microscope imaging.

### 2.8 Cellular mitochondrial membrane potential (MMP) staining

The treated cells were added to the prepared Mito-Tracker Deep Red FM (Beyotime, C1032) and incubated at 37 °C for 15–30 min. At the end of the staining, fresh cell culture medium pre-warmed at 37 °C was added. Place under fluorescence microscope for observation.

### 2.9 Statistical analysis

All experimental data were analyzed by three independent tests and expressed as mean ± standard deviation (x ± s) using SPSS 17.0 software. Comparisons among groups were made by one-way ANOVA followed by Tukey’s *post hoc* test. A P-value <0.05 was considered statistically significant.

## 3 Results

### 3.1 Cytotoxicity of 10-HDA and LPS in RAW264.7 cells

**FIGURE 1:**
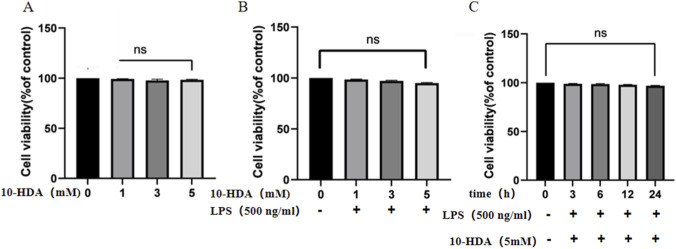
10-HDA and LPS do not affect RAW264.7 cell viability. **(A)** Effect of 10-HDA treatment on cell viability at different doses. **(B)** Effect of LPS and 10-HDA treatment on cell viability. **(C)** Effect of LPS+10-HDA treatment on cell viability at different times.

Cytotoxicity assay was determined by CCK8 method. As shown in Figure 1A, the cell viability was almost unaffected after treatment with 10-HDA (1 mM, 3 mM, 5 mM).

The cell viability was not significantly affected after treatment, as shown in Figure 1B. The results of pretreatment with 500 ng/mL of LPS for 1 h followed by treatment with 10-HDA (1 mM, 3 mM, 5 mM) for 24 h. As shown in Figure 1C, treatment with 500 ng/mL LPS and 5 Mm 10-HAD at different times (0, 3, 6, 12, and 24 h) showed that the cell viability was not significantly affected after treatment. The results indicate that 10-HDA doses at 0–5 mM are fully compliant with standard operation and do not produce significant cytotoxicity under the treatment methods and times used in this experiment. Therefore, in all the following experiments, the treatment regimen of pretreatment with 500 ng/mL of LPS for 1 h followed by treatment with 10-HDA for 12 h or 24 h was followed. Based on the preliminary viability results, the 3 mM concentration, which showed an effect similar to that of 1 mM, was excluded from subsequent experiments to focus on representative low (1 mM) and high (5 mM) doses.

### 3.2 10-HDA attenuates the transformation of RAW264.7 to M1 type

**FIGURE 2:**
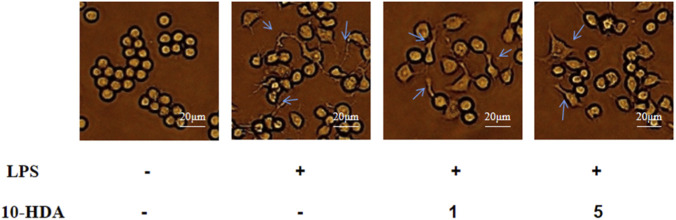
The effect on RAW264.7 cell morphology and viability of 10-HDA treatment. Phase contrast images of RAW264.7 cells untreated or treated with LPS or LPS/10-HDA (1 mM, 5 mM).

To preliminarily assess the effect of 10-HDA on M1 polarization, we examined cell morphology. As shown in Figure 2, under control conditions (normal culture), RAW264.7 cells exhibited a typical spherical morphology with a smooth cell membrane. Following LPS stimulation (500 ng/mL), which induces M1 polarization, the cells underwent a dramatic morphological transformation, becoming elongated, spindle-shaped, or amoeboid, accompanied by the extension of numerous filopodia and lamellipodia. Treatment with 10-HDA (1 and 5 µM) during LPS challenge dose-dependently attenuated these changes. Specifically, the number and length of filopodia were reduced, fewer cells displayed irregular shapes, and some cells reverted to a spherical or oval morphology. Notably, at the highest dose of 10-HDA (5 µM), most cells presented a near-spherical shape with minimal membrane protrusions. These observations indicate that 10-HDA can effectively inhibit the LPS-induced morphological transformation of RAW264.7 cells toward the M1 phenotype.

### 3.3 10-HDA attenuates LPS-induced cytokine in RAW264.7 cells

**FIGURE 3:**
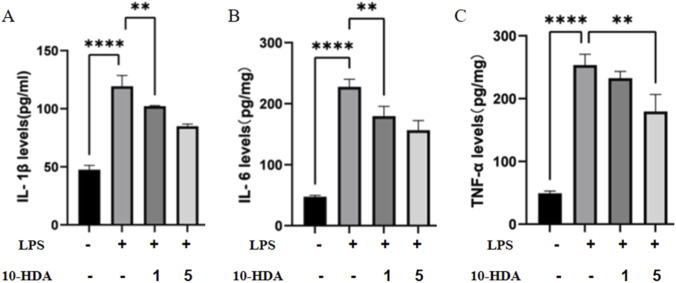
Inhibitory effect of 10-HDA on LPS-induced production of pro-inflammatory cytokines. RAW264.7 cells were incubated with LPS (500 ng/mL) for 24 h, or LPS for 1 h followed by 10-HDA treatment (1, 5 mM) for 24 h (with LPS still in the medium), release of the proinflammatory cytokines, IL-1β **(A)** IL-6 **(B)** and TNF-α **(C)** into the supernatants was examined by ELISA method. Data are expressed as mean ± SD (n = 3; *P < 0.05, **P < 0.01, ***P < 0.001).

To evaluate the potential anti-inflammatory effect of 10-HDA, the RAW264.7 was incubated with LPS for 1 h and then treated with 10-HDA (1 mM, 5 mM) for 24 h. Changes in pro-inflammatory cytokines IL-1β, IL-6 and TNF-α were detected using ELISA. The ELISA results showed that stimulation of RAW264.7 with LPS significantly increased the production of pro-inflammatory production of pro-inflammatory cytokines IL-1β, IL-6 and TNF-α. However, when 10-HDA was added, the levels of these cytokines decreased in a dose-dependent manner, suggesting that 10-HDA inhibits LPS-induced pro-inflammatory responses in RAW264.7 (Figures 3A–C).

### 3.4 10-HDA affects M1, M2 type macrophage markers and the content of IL-1β, COX-2 in mRNA level

**FIGURE 4:**
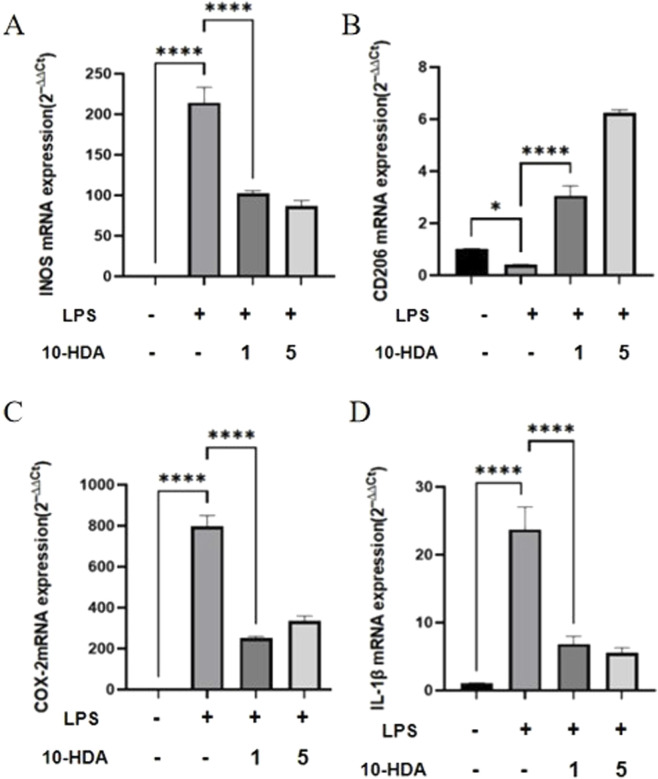
10-HDA affects LPS-induced inflammation and polarization-related mRNA expression levels in RAW264.7 cells. RAW264.7 cells were incubated with LPS (500 ng/ml) alone, or LPS for 1 h followed by 10-HDA treatment (1, and 5 mM) for 24 h, mRNA level of M1, M2 type macrophage markers and the changes in the content of IL-1β, COX-2 was measured by RT-PCR. **(A)** The levels of M1 phenotypic markers iNOS. **(B)** The levels of M2 phenotypic markers CD206. **(C,D)** The content of COX-2 and IL-1β in mRNA level. The data were generated from three independent experiments. Data are expressed as mean ± SD (n = 3; *P < 0.05, **P < 0.01, ***P < 0.001).

In this experiment, real-time PCR was used to detect the mRNA expression of M1, M2 type macrophage markers and the changes in the content of IL-1β, COX-2, as shown in the Figures 4A,C,D, in LPS-stimulated RAW264.7 cells, the levels of M1 phenotypic markers, iNOS mRNA, COX-2 mRNA and IL-1β mRNA, were significantly increased; iNOS mRNA,COX-2 mRNA and IL-1β mRNA expression was inhibited by the intervention of 10-HDA, but the level of CD206 was significantly elevated (Figure 4B). The differences of iNOS, CD206, COX-2 and IL-1β were statistically significant compared with the control group and the experimental group.

### 3.5 10-HDA affects M1, M2 type macrophage markers and the content of IL-1β, COX-2 in protein level

**FIGURE 5:**
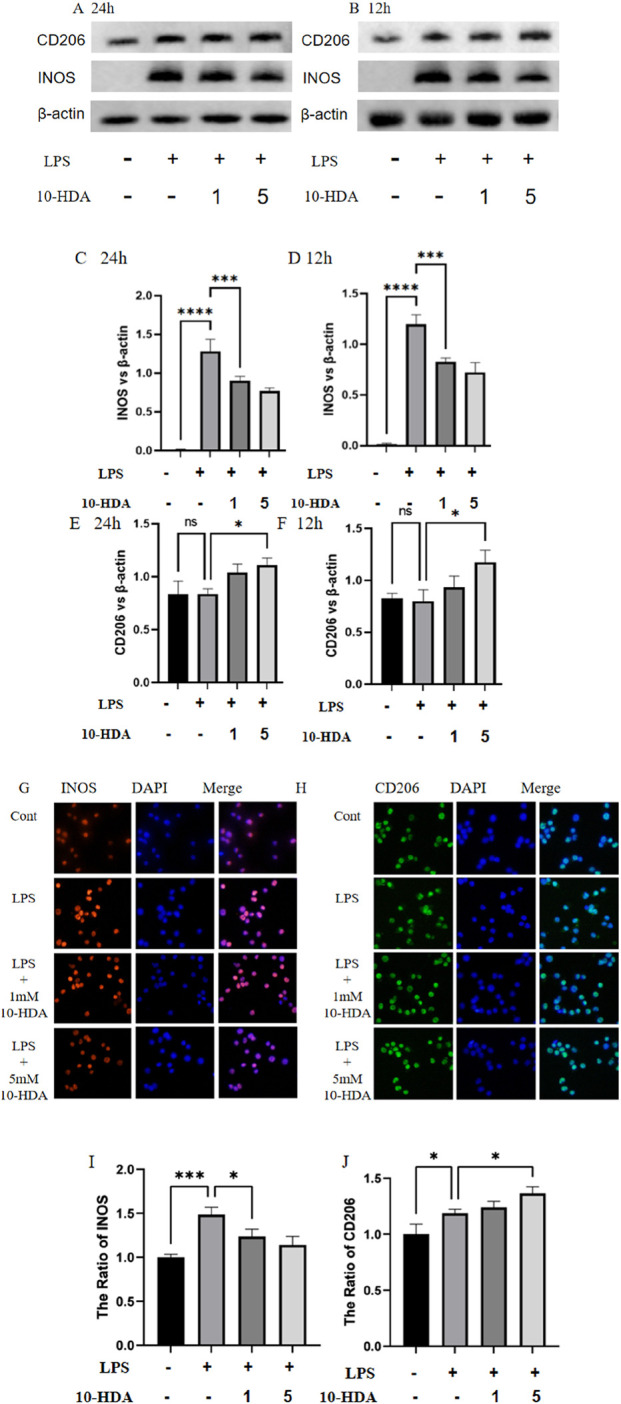
10-HDA affects LPS-induced polarization-related protein expression levels in RAW264.7 cells. RAW264.7 cells were incubated with LPS (500 ng/ml) alone, or LPS for 1 h followed by 10-HDA treatment (1 and 5 mM) for 12 h, 24 h, protein level of M1(iNOS), M2(CD206) type macrophage markers were measured by Western blotting assay. **(A)** Representative western blot analyses of iNOS and CD206 for 24 h. **(B)** for 12 h. **(C)** Bands were analyzed and quantified by densitometry and the iNOS /β-actin ratio was evaluated for 24 h. **(D)** Bands were analyzed and quantified by densitometry and the iNOS/β-actin ratio was evaluated for 12 h. **(E)** Bands were analyzed and quantified by densitometry and the CD206/β-actin ratio was evaluated for 24 h. **(F)** Bands were analyzed and quantified by densitometry and the CD206/β-actin ratio was evaluated for 12 h. **(G,H)** Representative cell immunofluorescence assay of iNOS and CD206 for 24 h. DAPI was used for nuclei staining. **(I,J)** Bands were analyzed and quantified by densitometry was evaluated. Data are expressed as mean ± SD (n = 3; *P < 0.05, **P < 0.01, ***P < 0.001).

Western blotting assay showed downregulation of M1-type macrophage markers and upregulation of the expression of M2 macrophage markers promoted M2 polarization. Compared with the control group, the expression of M1-type marker iNOS was significantly higher in the LPS group, and the iNOS content was significantly lower in the LPS+1mM10-HDA group and LPS+5mM10-HDA group compared with that of the LPS group, showing a concentration correlation (Figures 5A–D), and there was no significant difference in the iNOS content at 12 h compared with that at 24 h (Figures 5A–D); The M2 type marker CD206 in the LPS+1 and 5mM10-HDA group was significantly higher than that in the control group and the LPS group (Figures 5A,B,E,F), and there was a correlation between the levels and concentrations, and there was no significant difference in the expression of CD206 after 12 h versus 24 h of 10-HDA treatment (Figures 5A,B,E,F). RAW264.7 cells were incubated with LPS (500 ng/mL) alone, or LPS for 1 h followed by 10-HDA treatment (1 and 5 mM) for 24 h. The results of immunofluorescence assay showed that LPS decreased the expression of iNOS and CD206 protein, whereas the expression of iNOS and CD206 protein was significantly increased after the addition of 10-HDA intervention with concentration correlation (Figures 5G–J).

### 3.6 10-HDA attenuates the expression of inflammation-related proteins in RAW264.7 cells

**FIGURE 6:**
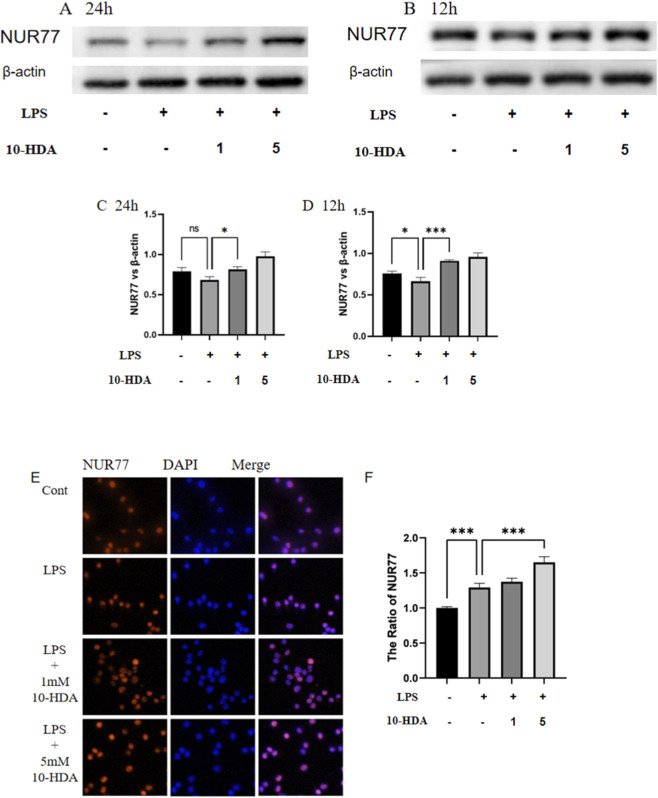
10-HDA upregulates NUR77 protein expression in RAW264.7 cells. RAW264.7 cells were incubated with LPS (500 ng/mL) alone, or LPS for 1 h followed by 10-HDA treatment (1 and 5 mM) for 12 h, 24 h, protein level of NUR77 was measured by Western blotting and cell immunofluorescence assay, **(A)** Representative Western blot analyses of NUR77 and β-actin for 24 h **(B)** for 12 h. **(C)** Bands were analyzed and quantified by densitometry and the NUR77/β-actin ratio was evaluated for 24 h. **(D)** Bands were analyzed and quantified by densitometry and the NUR77/β-actin ratio was evaluated for 24 h. **(E)** Representative cell immunofluorescence assay of NUR77 for 24 h. DAPI was used for nuclei staining. **(F)** Bands were analyzed and quantified by densitometry was evaluated. Data are expressed as mean ± SD (n = 3; *P < 0.05, **P < 0.01, ***P < 0.001).

The results of Western blotting method and immunofluorescence assay showed that LPS decreased the expression of NUR77 protein, whereas the expression of NUR77 protein was significantly increased after the addition of 10-HDA intervention with concentration correlation (Figures 6A–F).

### 3.7 Effects of 10-HDA on NF-κB signaling pathways

**FIGURE 7:**
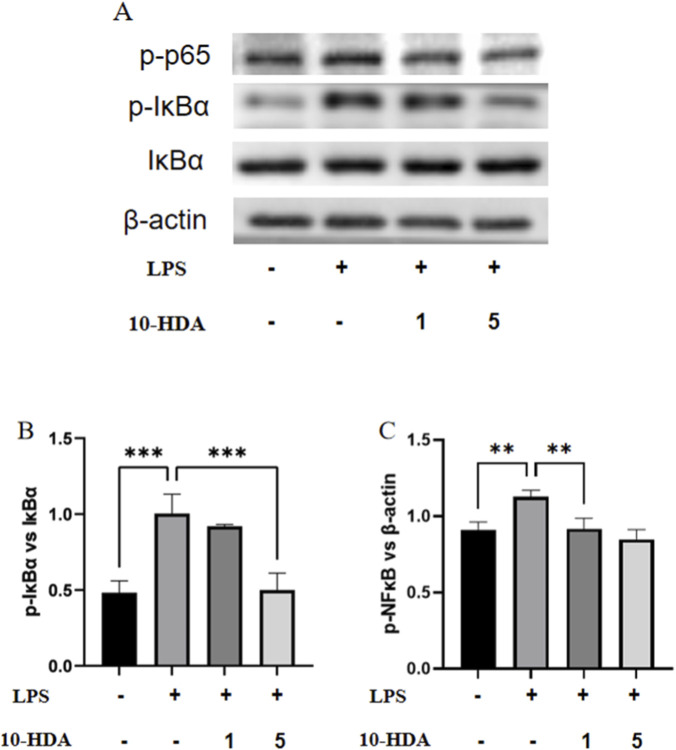
Effects of 10-HDA on NF-κB nuclear translocation in RAW264.7 exposed to LPS. **(A)** Representative western blot analyses of NF-κB p65, IκBα, p-IκBα and β-actin. **(B)** Bands were analyzed and quantified by densitometry and the p-IκBα/ IκBα ratio was evaluated. **(C)** Bands were analyzed and quantified by densitometry and the NF-κBp65/β-actin ratio was evaluated. Data are expressed as mean ± SD (n = 3; *P < 0.05, **P < 0.01, ***P < 0.001).

The results were shown in Figures 7A–C, after treatment with 500 ng/mL LPS, the NF-κB signaling pathway related proteins p-NF-κB, p-IκBα were all activated rapidly, and the addition of 10-HDA significantly inhibited the downregulation of the phosphorylation of NF-κB, and p-IκBα was more obvious inhibited by 10-HDA, compared with that of the LPS group.

### 3.8 10-HDA upregulates the mitochondrial activity in RAW264.7 cells

**FIGURE 8:**
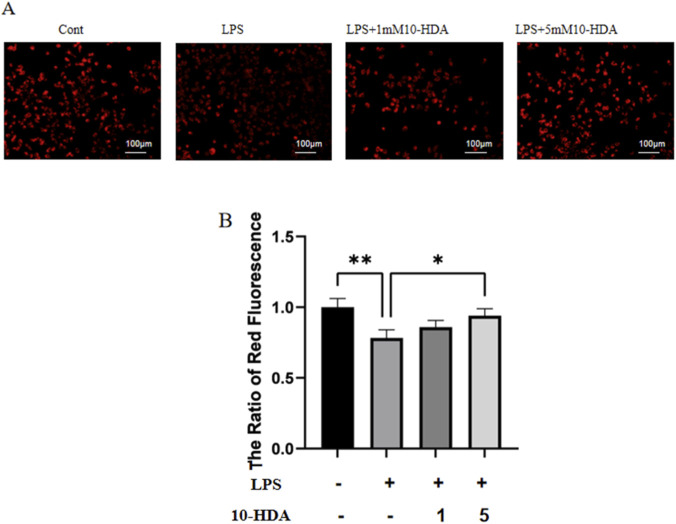
Mitochondrial membrane potential (MMP) staining results. RAW264.7 cells were incubated with LPS (500 ng/mL) alone, or LPS for 1 h followed by 10-HDA treatment (1 and 5 mM) for 24 h. **(A)** Detection of MMP. **(B)** Bands were analyzed and quantified by densitometry was evaluated. Data are expressed as mean ± SD (n = 3; *P < 0.05, **P < 0.01, ***P < 0.001).

From the results of mitochondrial staining in Figures 8A,B, it can be seen that the RAW264.7 group showed normal red fluorescence, and after the intervention of adding LPS, the fluorescence brightness was obviously reduced, the fluorescence outline was not clear, and the color was a little darker, and after the addition of 10-HDA, the fluorescence brightness was enhanced, and the fluorescence range increased, which was significantly different from that of the LPS group, and it can be obtained that after the addition of 10-HDA, the effect of LPS on the mitochondria can be attenuated, and the effects of LPS can be restored partially to the damage to mitochondria.

### 3.9 10-HDA modulates LPS-stimulated metabolic effects in mitochondria of RAW264.7 cells

**FIGURE 9:**
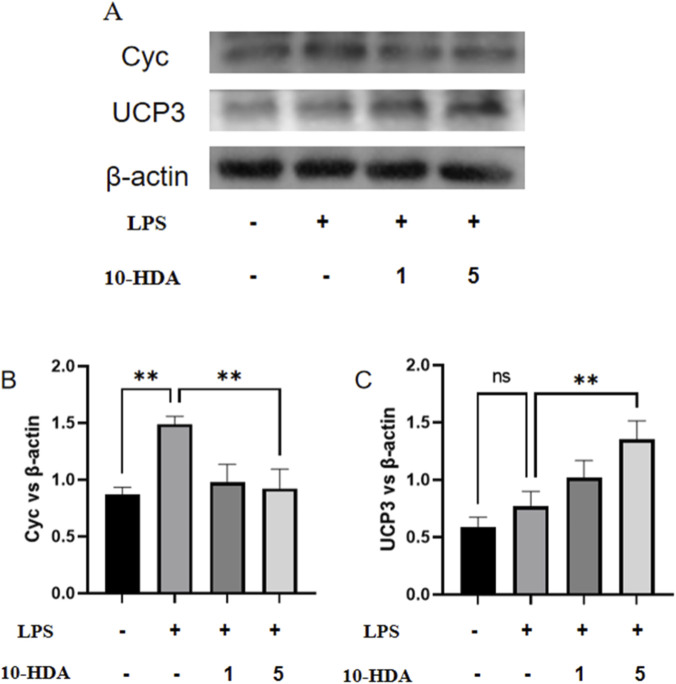
Effect of 10-HDA on protein expression of mitochondrial metabolism-related proteins Cyc, Ucp3 in LPS-induced RAW264.7. RAW264.7 cells were incubated with LPS (500 ng/mL) alone, or LPS for 1 h followed by 10-HDA treatment (1 and 5 mM) for 24 h **(A)** protein level of Cyc and UCP3 were measured by Western blotting. **(B,C)** Bands were analyzed and quantified by densitometry and ratio was evaluated. Data are expressed as mean ± SD (n = 3; *P < 0.05, **P < 0.01, ***P < 0.001).

Compared with the control group, RAW264.7 stimulated by LPS showed a significant elevation of Cyc, and the effect of LPS on Cyc was significantly reduced by the addition of 10-HDA, and the UCP3 protein was gradually elevated with the increase of 10-HDA, indicating that it led to significant mitochondrial metabolism changes in RAW264.7 cells, which was attenuated by 10-HDA (Figures 9A–C).

## 4 Discussion

Royal jelly is a yellowish-white acidic secretion from the hypopharyngeal and mandibular glands of worker bees, which is widely used in medicine and healthcare due to its unique physiological effects (Melliou and Chinou, 2005). 10-hydroxy-2-decenoic acid (10-HDA), one of the markers of royal jelly, is a unique fatty acid, or rosmarinic acid, that exists exclusively in royal jelly (Honda et al., 2015). As a natural product derived from royal jelly, 10-HDA is generally recognized as safe in dietary contexts; however, a rigorous evaluation of its potential side effects was beyond the scope of this *in vitro* study. Previous studies have shown that 10-HDA has a strong inhibitory effect on a variety of bacteria, such as *Staphylococcus aureus*, Enterobacteriaceae, and *Bacillus subtilis* (Gao et al., 2022; Šedivá et al., 2018; Yang et al., 2018; Nazem et al., 2025), and enhances autoimmune and immunomodulatory activities (Dzopalic et al., 2011; Fan et al., 2020; Eslami-Kaliji et al., 2021; You et al., 2020). Scholars have demonstrated that 10-HDA has a good exogenous and *ex vivo* anti-inflammatory effect, and suggested that it can inhibit the release of the main inflammatory mediators, although the specific mechanism is still not clear. The concentration dependence of 10-HDA on the release of inflammatory mediators and NO was significant (Chen et al., 2018). At the same time, 10-HDA inhibited the expression of key inflammatory mediators, such as IL-6 and IL-10 (You et al., 2020). In addition, 10-HDA also has anti-colitis (Huang et al., 2022), anti-aging (Mirbaha et al., 2019; Park et al., 2011), anti-tumor (Tian et al., 2024; Saad Al Shehri et al., 2023; Albalawi et al., 2021; Lin et al., 2020; Botezan et al., 2023), antioxidant (Han et al., 2023), 10-HDA has a variety of biological activities such as antihypertensive (Cai et al., 2018). 10-HDA is of great importance as a potential anti-inflammatory drug.

In the present study, RAW 264.7 macrophages were selected as the effector cells in an *in vitro* inflammatory model to investigate the anti-inflammatory effects of 10-HDA *in vitro*. Firstly, the activity of LPS,10-HDA on RAW 264.7 macrophages was detected by CCK-8 assay. The results showed that LPS at 500 ng and 10-HDA in the range of (0–5 mM) had no effect on the cell viability. We then confirmed the inhibitory effect of 10-HDA on the expression of inflammatory factors in macrophages. We then explored the possible mechanisms by which 10-HDA inhibits the inflammatory response. A large number of studies have demonstrated that inducing M2 polarization in macrophages can achieve anti-inflammatory effects. For example, the active ingredients of Celastrus orbiculatus Thunb, Hyperoside, Tetrandrine, Pulsatilla Saponins, Viola yedoensis Makino formula, and Dandelion can induce M2 polarization of macrophages to exert anti-inflammatory effects (Zhang et al., 2025; Zhao et al., 2025; Fang et al., 2024; Yang et al., 2023; Zeng et al., 2022; Li et al., 2022). In the present study, we found that 10-HDA significantly downregulated the M1 macrophage marker iNOS and upregulated the M2 macrophage marker CD206, and we hypothesized that 10-HDA could play an anti-inflammatory role by regulating macrophage polarization.

NUR77, a member of the NR4A subfamily of nuclear receptors, has been shown to be a key regulator of the inflammatory response and its downstream pathway, the NF-κB pathway (Liu-Bryan and Terkeltaub, 2015). NUR77 can be activated via the NF-κB pathway, and activated NUR77 can inhibit NF-κB activation by disrupting p65 binding to DNA (Shaked et al., 2015). NUR77 is a natural regulator of the inflammatory response in physiological conditions, and inhibits inflammation by disrupting p65 binding to DNA, which provides reference value in inhibiting inflammation and ameliorating inflammatory diseases (Rodríguez-Calvo et al., 2017). In our study, results show that 10-HDA reduces NUR77 expression and is associated with decreased markers of M1 polarization. However, given the exploratory nature of this study and the complex bioactive properties of 10-HDA, the involvement of other signaling or bio-energetic pathways cannot be ruled out. Further studies are needed to determine whether NUR77 plays a causal role or if other mechanisms (e.g., metabolic reprogramming) are primarily involved. There was no difference in NUR77 expression levels between 12 h and 24 h. Current studies have shown that NUR77 is a natural negative feedback regulator of the NF-κB signaling pathway, and it can inhibit the expression of genes related to inflammation in chondrocytes, as NUR77 inhibits the transcriptional ability of p65 and prevents IL-1B-induced expression of matrix metalloproteinases in chondrocytes, suggesting that NUR77 has a role to play in bone and joint inflammation. Meanwhile, our research showed that the protein expression levels of both p-NF-κB and p-IκBα were decreased under 10-HDA. The pathway of the NUR77/NF-κB signaling was regulated under 10-HDA.

Mitochondria are the only independent organelles with their own double-stranded circular DNA. The basic function of mitochondria is to provide energy for cellular life activities, and the interaction between the oxidative phosphorylation (OXPHOS) system and the mitochondrial quality control system maintains the normal function of mitochondria, such as ATP production and the elimination of reactive oxygen species (ROS), etc. The mitochondria can produce energy while releasing a large amount of ROS (Palma et al., 2024; Lee and Paull, 2020; Lenaz, 2012). Abnormal mitochondrial metabolism leads to a decrease in ATP production and an increase in ROS content, and the oxidative stress caused by elevated ROS results in changes in the structure and function of mitochondria, and the mitochondrial metabolism abnormality also triggers mitochondrial autophagy and overloading of the calcium ion concentration, resulting in cellular damage. Cyc is a key electron transporter in mitochondria that promotes cellular oxidative phosphorylation, provides energy to the organism, and manifests its oxidative metabolism in mitochondria. Mitochondrial UCP3 is transcribed and translated from the nucleus, regulated by the mitochondrial transporter enzyme complex TOM/TIM, and transcribed to the mitochondrial membrane to perform its biological functions. UCP3 has a slight decoupling effect on mitochondrial membrane potential and reduces reactive oxygen species (ROS). UCP3 has a slight decoupling effect on the mitochondrial membrane potential and reduces the generation of reactive oxygen species (ROS). UCP3 can also reduce or inhibit the generation of ROS through various ways, such as direct activation of mitochondrial glutathione peroxidase and lowering the electrical pressure of the mitochondrial membrane, etc. UCP3 plays an important role in maintaining the efficiency of the energy conversion of the mitochondria, and in protecting the function of the mitochondria (Sánchez-Pérez et al., 2023; Fortes et al., 2023; Codella et al., 2023; Chen et al., 2021).

While our data show that 10-HDA upregulates UCP3 and reduces mitochondrial ROS levels, we acknowledge that this does not rule out direct actions of the compound on macrophage redox pathways. In particular, macrophages possess robust inducible antioxidant response elements (e.g., Nrf2/ARE) and NADPH oxidase-derived ROS used for pathogen killing. A reduction in mitochondrial ROS does not necessarily impair the phagocytic respiratory burst; instead, it may enhance overall cell viability or prevent chronic oxidative damage. Future work should determine whether [bioactive agent] directly activates Nrf2, alters NOX2 activity, or modulates UCP3 expression independently of mitochondrial uncoupling. Our current results are therefore best interpreted as evidence of improved mitochondrial homeostasis, which may complement, rather than compromise, macrophage effector functions.

The above studies suggest that 10-HDA may exert its anti-inflammatory effects *in vitro* by regulating the expression of proteins related to the NUR77/NF-κB signaling pathway, affecting macrophage polarization and regulating mitochondrial metabolism.

A potential limitation of this study is the use of high-glucose DMEM for cell culture. Several studies have demonstrated that high-glucose conditions can promote M1-like polarization of RAW264.7 macrophages and contribute to mitochondrial dysfunction. However, because all groups were cultured under identical conditions, the comparative conclusions regarding the anti-inflammatory effect of 10-HDA remain robust. The uniform culture environment ensures that any high-glucose-induced bias, if present, is equally distributed across groups, leaving the relative effect sizes and statistical comparisons between groups unaffected. Given the dynamic nature of macrophage polarization, it is important to note that the observed M1-suppressing effect of 10-HDA may be reversible. Removal of 10-HDA from the culture media might allow macrophages to reacquire a full M1 phenotype upon re-stimulation, which warrants further investigation in future studies.

We acknowledge several limitations. First, the absence of M2-polarizing positive controls (e.g., IL-4) means we cannot conclusively claim that 10-HDA actively promotes canonical M2 polarization. Second, our data do not include direct bio-energetic measures (e.g., extracellular flux analysis for OCR/ECAR); therefore, we refrain from concluding any metabolic reprogramming. Our results should be interpreted as suggestive evidence that 10-HDA can temper M1-associated features, with a gene expression shift that partially resembles an M2-like state. Future studies employing metabolic inhibitors, M2-specific reporters, and comprehensive bio-energetic assays are required to validate the intriguing hypothesis raised by our findings. Meanwhile, our results demonstrate anti-inflammatory effects of 10-HDA at the cellular level, further studies in animal models are required to confirm its activity within the complex physiological environment of systemic inflammation.

## Data Availability

The original contributions presented in the study are included in the article. Further inquiries can be directed to the corresponding author.
